# Correlation of clinical, laboratory, and short‐term outcomes of immunocompromised and immunocompetent COVID‐19 patients with semi‐quantitative chest CT score findings: A case‐control study

**DOI:** 10.1002/iid3.1239

**Published:** 2024-04-05

**Authors:** Abdolkarim Haji Ghadery, Ladan Abbasian, Fatemeh Jafari, Niloofar Ayoobi Yazdi, Zahra Ahmadinejad

**Affiliations:** ^1^ Department of Radiology, Advanced Diagnostic and Interventional Radiology Research Center(ADIR) Tehran University of Medical Sciences Tehran Iran; ^2^ Iranian Research Center for HIV/AIDS, Iranian Institute for Reduction of High Risk Behaviors, Department of Infectious Diseases, Imam Khomeini Hospital Complex, School of Medicine Tehran University of Medical Sciences Tehran Iran; ^3^ Department of Radiology, Advanced Diagnostic and Interventional Radiology Research Center (ADIR) Tehran University of Medical Sciences Tehran Iran; ^4^ Department of Infectious Diseases, Liver Transplantation Research Center, Imam Khomeini Hospital Complex Tehran University of Medical Sciences Tehran Iran

**Keywords:** case‐control, chest CT score, COVID‐19, immunocompromised, immunocompetent

## Abstract

**Background:**

As the effects of immunosuppression are not still clear on COVID‐19 patients, we conducted this study to identify clinical and laboratory findings associated with pulmonary involvement in both immunocompromised and immunocompetent patients.

**Methods:**

A case‐control of 107 immunocompromised and 107 immunocompetent COVID‐19 patients matched for age and sex with either positive RT‐PCR or clinical‐radiological findings suggestive of COVID‐19 enrolled in the study. Their initial clinical features, laboratory findings, chest CT scans, and short‐term outcomes (hospitalization time and intensive care unit [ICU] admission) were recorded. In addition, pulmonary involvement was assessed with the semi‐quantitative scoring system (0−25).

**Results:**

Pulmonary involvement was significantly lower in immunocompromised patients in contrast to immunocompetent patients, especially in RLL (*p* = 0.001), LUL (*p* = 0.023), and both central and peripheral (*p* = 0.002), and peribronchovascular (*p* = 0.004) sites of lungs. Patchy (*p* < 0.001), wedged (*p* = 0.002), confluent (*p* = 0.002) lesions, and ground glass with consolidation pattern (*p* < 0.001) were significantly higher among immunocompetent patients. Initial signs and symptoms of immunocompromised patients including dyspnea (*p* = 0.008) and hemoptysis (*p* = 0.036), respiratory rate of over 25 (*p* < 0.001), and spo2 of below 93% (*p* = 0.01) were associated with higher pulmonary involvement. Total chest CT score was also associated with longer hospitalization (*p* = 0.016) and ICU admission (*p* = 0.04) among immunocompromised patients.

**Conclusions:**

Pulmonary involvement score was not significantly different among immunocompromised and immunocompetent patients. Initial clinical findings (dyspnea, hemoptysis, higher RR, and lower Spo_2_) of immunocompromised patients could better predict pulmonary involvement than laboratory findings.

## 1 INTRODUCTION

On December 31, 2019, a novel coronavirus was isolated from the respiratory secretions of numerous individuals in Wuhan, China, who were suffering from an unexplained lower respiratory tract infection.^1^ Because of the highly contagious nature of coronavirus disease 2019 (COVID‐19), the World Health Organization declared it a pandemic on March 11, 2020.

It is still a controversial subject if immunocompromised patients are at increased risk for severe COVID‐19 outcomes than their immunocompetent counterparts, as some studies suggest immunocompromised patients are at increased risk of severe complications due to reduced immune defenses caused by the underlying disease or therapy.^2^, ^3^ However, due to the link between COVID‐19 and high levels of cytokine production,^4^ immunosuppression may dampen the infection's exuberant inflammatory response. Previous studies reported either no differences between severity and adverse outcomes of COVID‐19 among immunocompromised and nonimmunocompromised patients^5^ or lower risk of severe outcomes in immunocompromised patients.^6^ A review article by Fung et al.^7^ reported solid organ recipients and human immunodeficiency virus (HIV) patients might be at increased risk of severe COVID‐19 disease or death, while the effects of other causes of immunodeficiency on COVID‐19 patients were not clear.

Chest CT scoring system was developed early in the COVID‐19 pandemic to objectively assess the extent of pulmonary involvement.^8^ It was later demonstrated that this scoring system correlates with and predicts with disease severity and prognosis of COVID‐19 patients.^9^, ^10^

**Figure 1:**
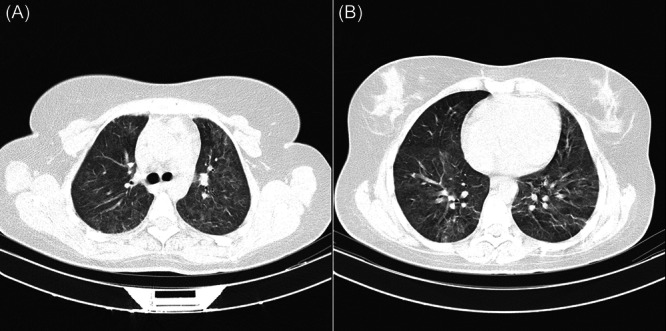
Chest CT Scan of an immunocompromised patient with diffuse involvement of the lung. These pictures, show 34‐year‐old immunocompromised female patient with diffuse involvement of all five lobes of her lung, this pattern was significantly higher among immunocompromised patients. All 5 lobes of this patient's lung (Shown in A and B) scored 5/5 based on the semiquantitative scoring system with a sum of 25/25 showing the severity of lung involvement in this patient.

**Figure 2:**
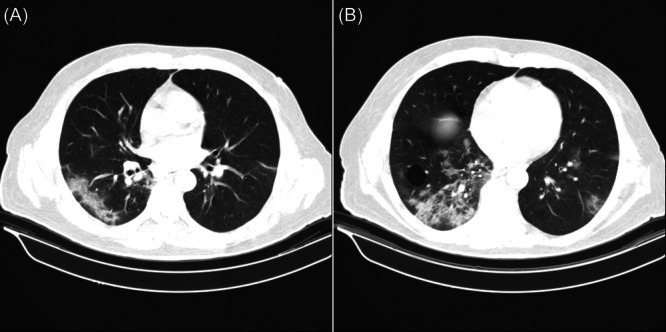
Chest CT scan of an immunocompetent patient with ground glass and consolidation pattern of lesions. These Pictures, depict a 54‐year‐old immunocompetent male patient with involvement of Right lower and left lower lobes, with central, peripheral, and anteroposterior locations of lesions, and both wedged and confluent shapes of lesions and mixed patterns of ground glass and consolidation which were significantly higher among immunocompetent patients. The right lower lobe scored 4/5 and the left lower lobe scored 3/5 (Shown in A and B) based on the semiquantitative scoring system and with 1/5 for the other three lobes with the sum of 10/25.

Here, we conducted a case‐control study for hospitalized immunocompromised and immunocompetent COVID‐19 patients and discussed their initial signs and symptoms, laboratory and imaging findings among these patients to understand which clinical or Para clinical findings on immunocompromised COVID‐19 patients should be prioritized to estimate the pulmonary involvement in these patients and answer this question if immunocompromised patients are at increased risk to be affected by COVID‐19 virus by comparing their lung involvement scores with their immunocompetent counterparts (Figures 1 and 2).

## 2 PATIENTS AND METHODS

### 2.1 Study design and participants

This study is a case‐control, Retrospective, single‐center study conducted in June and July 2020 at Imam Khomeini Hospital Complex (a tertiary care educational hospital), in Tehran, Iran. All hospitalized COVID‐19 cases were included, and patients were selected consecutively, with either definitive (based on positive polymerase chain reaction of respiratory secretions) or probable (based on clinical symptoms such as sore throat, cough, myalgia, weakness, lethargy, sweating, shortness of breath, diarrhea, fever, and radiological findings such as lung involvement in a chest CT scan or chest X‐ray) cases. COVID‐19 patients with an immune deficiency were grouped as cases, and immunocompetent patients were grouped as controls. The hospital records of 214 patients with COVID‐19 infection were reviewed (107 immuno‐deficient and 107 immunocompetent patients matched for age and sex).

The case group consisted of immunocompromised patients who matched the following criteria:

- A corticosteroid dose of more than 10 mg for more than 3 weeks.
- Low‐dose corticosteroids for over 6 months with a cumulative dose of 750 mg or higher.
- Other immunosuppressive or cytotoxic medicines used in the last 6 months, such as azathioprine, methotrexate, mycophenolate mofetil (Cellcept), tacrolimus (Prograf), anti‐TNF alpha, and other biologics.
- Transplantation of a solid organ or bone marrow.
- Hematologic cancers that were treated with or without chemotherapy in the previous 6 months.
- Chemotherapy for solid organ cancers in the previous 6 months.
- Infection with the HIV/acquired immunodeficiency syndrome
- Primary immunodeficiencies, such as chronic granulomatous disease and common variable immune deficiency.

The patients without previous conditions were grouped as immunocompetent patients.

### 2.2 Data collection

Each patient's clinical data were collected on separate forms, which included: (a) demographic information (age and gender); (b) vital signs: temperature (T‐degrees Celsius), oxygen saturation (SpO_2_), respiratory rate (RR/min), and pulse rate (PR/min); (c) signs and symptoms: fever, sweating, myalgia, dyspnea, nausea‐vomiting, hemoptysis, anosmia; (d) comorbidities such as hypertension (HTN) and diabetes mellitus (DM), smoker, chronic kidney disease (CKD), ischemic heart disease (IHD); (e) laboratory data: white blood cell (WBC) count (cell/mm^3^), lymphocyte count (cell/mm^3^), platelet count (cell/mm^3^), C‐reactive protein (CRP‐mg/L), erythrocyte sedimentation rate (ESR‐mm/h), and lactate dehydrogenase (LDH) (units/L), aspartate transaminase (AST) (units/L), alanine transaminase (ALT) (units/L); (f) intensive care unit (ICU) admission; (h) radiologic abnormalities (discussed in the following section); (i) length of hospitalization (day).

### 2.3 Chest CT protocols and interpretations

With patients in the supine position and full inspiration breath‐hold(within the patient's maximum ability), all chest CT scans were done on a lightspeed 64‐detector CT (GE Health care) from the apex to the lung base. The following were the most significant scanning parameters: 120 kVp tube voltage; 50−150 mAs tube current; 0.75‐s tube rotation time; 0.5–0.75‐s gantry rotation time; 2–3‐mm section thickness; and 0.6–2 mm beam collimation.

Two thoracic radiologists with 15 and 20 years of expertize in consensus performed the visual chest CT interpretation. The radiologists were blinded to the participants' clinical and laboratory data and examined lung and mediastinal window settings. The presence of CT features such as the following were reported: (a) Axial location of lesion: Central, Peripheral, both Central and peripheral, Peribronchovascular, or diffuse; (b) Anteroposterior location of lesions: Anterior, Posterior, both Anterior and posterior, and Diffuse; (c) the dominant type of lesions: patchy, nodular, reticular, or diffuse; (d) the dominant shape of lesions: round, elongated, wedged, confluent, or diffuse; (e) the predominant pattern of lesions: ground‐glass opacification (GGO), consolidation, the combination of GGO and consolidation, Crazy paving pattern, Cavitation, Solid, Part‐solid, and Reverse‐ halo.

A semi‐quantitative CT severity grading was established by Yang et al.^8^ and Pan et al.^11^ were used to determine the amount of pathological involvement: each of the five lobes of the lungs was visually scored from 0 to 5 (0, no involvement; 1, 5–25% involvement; 3, 26–49% involvement; 4, 50–75% involvement; 5, >75% involvement). The overall chest CT score was then obtained by adding the scores of each lobe, which ranged from 0 to 25.

### 2.4 Statistical analysis

SPSS version 16 was used for the statistical analysis (SPSS Inc.). Continuous variables were represented by their mean (standard deviation), whereas categorical variables were represented by their frequency and percentages. The Kolmogorov−Smirnov test was used to determine the normality of the variables. The independent sample *t*‐test was used to evaluate normally distributed continuous variables; otherwise, the Mann−Whitney *U* test was utilized. For nominal variables, the Chi‐square test or Fisher's exact *t*‐test (if cases were lower than 5) was used. Statistical significance was defined as a *p* Value of less than .05.

## 3 RESULTS

### 3.1 Participant's characteristic

The current study included 107 immunocompromised COVID‐19 patients as the case group and 107 immunocompetent COVID‐19 patients as the control group. Participants were matched based on their age and gender. Of 107 immunocompromised patients, 14 (13.08%) had a solid organ transplant, 4 (3.73%) had HIV, 20 (18.69%) had hematologic malignancy, 37 (34.57%) had solid tumors, 5 (4.67%) had drug‐induced immunodeficiency, 5 (4.67%) had autoimmune disease, and 22 (20.56%) had a rheumatologic disease. The immunocompromised and immunocompetent COVID‐19 patients mean age was 54.43 (14.27) and 56.6 (14.72), respectively. 108 (50.5%) of all participants were male, and 54 (50.5%) of each group were also male.

### 3.2 Chest CT scan findings

A total of 170 patients had lung involvement from which RLL, 159 (74.29%) was the most affected lobe, and on the axial plane, lesions were mostly located on peribronchovascular site 101 (47.19%) and on both anterior and posterior sites of lungs, 130 (60.74%). The most common type of lesions were patchy lesions, 160 (74.76%), and confluent, 118 (55.14%) shape of lesions was the most common shape; meanwhile, the ground‐glass pattern, 138 (64.48) was the most prevalent pattern among patient's pulmonary lesions (Table 1).

**Table 1:** Radiologic findings of all, and both immunocompromised and immunocompetent patients.

		Groups		
Variables	All patients (*n* = 214)	Immunocompromised patients (*n* = 107)	Immunocompetent patients (*n* = 107)	*p* Value
Pulmonary involvement score				
RUL total score	2.95 (1.39)	2.91 (1.51)	2.98 (1.29)	0.74
RML total score	2.7 (1.63)	2.67 (1.78)	2.73 (1.51)	0.84
RLL total score	3.18 (1.39)	3.07 (1.53)	3.27 (1.28)	0.37
LUL total score	2.86 (1.43)	3.01 (1.52)	2.73 (1.35)	0.23
LLL total score	3.2 (1.37)	3.17 (1.48)	3.23 (1.27)	0.78
Total Pulmonary involvement score	13.07 (7.3)	13.2 (7.69)	12.96 (7.02)	0.83
Frequency of lobe involvement				
RUL	147 (68.69)	68 (63.55)	79 (73.83)	0.1
RML	131 (61.21)	59 (55.14)	72 (67.28)	0.68
RLL	159 (74.29)	69 (64.48)	90 (84.11)	0.001*, ^,a^
LUL	153 (71.49)	69 (64.48)	84 (78.50)	0.023*, ^,a^
LLL	152 (71.02)	70 (65.42)	82 (76.63)	0.071
Lung involvement	170 (79.43)	75 (70.09)	95 (88.78)	0.001*, ^,a^
Axial location of lesion				
Central	2 (0.93)	1 (0.93)	1 (0.93)	1
Peripheral	66 (30.84)	30 (28.03)	36 (33.64)	0.37
Central and peripheral	86 (40.18)	32 (29.9)	54 (50.46)	0.002*, ^,a^
Peribronchovascular	101 (41.19)	40 (37.38)	61 (57.0)	0.004*, ^,a^
Diffuse	14 (6.54)	11 (10.28)	3 (2.8)	0.027*, ^,a^
Anteroposterior location				
Anterior	3 (1.4)	0	3 (2.8)	0.08
Posterior	20 (9.34)	9 (8.41)	11 (10.28)	0.63
Anterior and posterior	130 (60.74)	57 (53.27)	73 (68.22)	0.02*, ^,a^
Diffuse	8 (3.73)	8 (7.47)	0	0.004*, ^,a^
Type of lesions				
Patchy	160 (74.76)	68 (63.55)	92 (85.98)	<0.001*, ^,a^
Nodular	19 (8.87)	12 (11.21)	7 (6.54)	0.22
Reticular	4 (1.86)	1 (0.93)	3 (2.8)	0.31
Diffuse	2 (0.93)	2 (1.86)	0	0.15
Shape of lesions				
Round	37 (17.28)	16 (14.95)	21 (19.62)	0.36
Elongated	41 (19.15)	17 (15.88)	24 (22.42)	0.22
Wedged	95 (44.39)	36 (33.64)	59 (55.14)	0.002*, ^,a^
Confluent	118 (55.14)	47 (43.92)	71 (66.35)	0.002*, ^,a^
Diffuse	6 (2.80)	6 (5.6)	0	0.013*, ^,a^
Pattern of lesion				
Ground glass	138 (64.48)	64 (59.81)	74 (69.15)	0.15
Consolidation	60 (28.03)	30 (28.03)	30 (28.03)	1
Ground glass and consolidation	99 (46.26)	34 (31.77)	65 (60.47)	<0.001*, ^,a^
Crazy paving pattern	43 (20.09)	23 (21.49)	20 (18.69)	0.35
Cavitation	1 (0.46)	1 (0.93)	0	‐
Solid	59 (27.57)	26 (24.29)	33 (30.84)	0.28
Part‐solid	41 (19.15)	17 (15.88)	24 (22.42)	0.21
Reverse‐halo	9 (4.20)	4 (3.73)	5 (4.67)	0.73

*Note*: Pulmonary involvement scores reported as mean (standard deviation), and all other variables reported as *N* (%).

Abbreviations: LLL, left lower lobe; LUL, left upper lobe; RLL, right lower lobe; RML, right middle lobe; RUL, right upper lobe.

* Statistically significant (*p* < 0.05).

^a^ Chi‐squared test or Fisher's exact *t*‐test is used; independent *t*‐test or Mann–Whitney *U* test used for all other comparisons.

Our results showed the overall pulmonary involvement scores and frequency of lesions were lower in immunocompromised patients rather than in immunocompetent patients. Immunocompetent patients had significantly higher RLL (*p* = 0.001), LUL (*p* = 0.023), and overall lung involvement (*p* = 0.001) compared to immunocompromised patients. Immunocompromised patients had significantly fewer lesions in both central and peripheral (*p* = 0.002) and peribronchovascular (*p* = 0.004) sites of lungs, while diffuse involvement of lungs in axial (*p* = 0.027) and sagittal (*p* = 0.004) plane was significantly higher among immunocompromised patients. Patchy (*p* < 0.001), wedged (*p* = 0.002), confluent (*p* = 0.002) lesions, and ground glass with consolidation pattern (*p* < 0.001) were significantly higher among immunocompetent patients (Table 1).

Further analysis showed total chest CT scores were associated with initial symptoms of immunocompromised patients, including dyspnea (*p* = 0.008) and hemoptysis (*p* = 0.036), while initial laboratory findings of immunocompetent patients were associated with total chest CT score. Total chest CT score among immunocompetent patients had a significant positive association with platelet counts with a cut point of 150,000 (mean of 9.50 ± 4.85 vs. 13.89 ± 7.24; *p* = 0.012), CRP with a cut point of 90 (mean of 10.56 ± 6.72 vs. 15.22 ± 6.58; *p* = 0.001), ESR with a cut point of 60 (mean of 15.22 ± 6.58 vs. 13.91 ± 7.02; *p* = 0.032), and AST with a cut point of 41 (mean of 10.92 ± 7.42 vs. 14.58 ± 6.29; *p* = 0.011). Both immunocompromised and immunocompetent patients had significantly higher total chest CT scores with an initial respiratory rate of over 25 (*p* < 0.001 and <0.0001, respectively) and spo_2_ of below 93% (*p* = 0.01 and <0.001, respectively).

Total chest CT score among immunocompromised patients was also associated with a higher risk of hospital stay (*p* = 0.016) and ICU admission (*p* = 0.04) (Table 2).

**Table 2:** Clinical, laboratory findings, and short term outcomes of both immunocompromised and immunocompetent patients based on the total chest CT score.

	Total CT score of immunocompromised patients	*p* Value	Total CT score of immunocompetent patients	*p* Value
Variables				
Symptoms on first visit				
Fever				
Yes	13.80 ± 7.49	0.325	12.87 ± 6.78	0.87
No	11.91 ± 8.11		13.10 ± 7.47	
Sweating				
Yes	16.55 ± 6.44	0.164	11.17 ± 7.51	0.24
No	12.74 ± 7.77		13.35 ± 6.89	
Myalgia				
Yes	12.67 ± 7.74	0.531	12.57 ± 6.91	0.44
No	13.80 ± 7.69		13.75 ± 7.26	
Dyspnea				
Yes	14.93 ± 8.09	0.008*, ^,a^	13.57 ± 7.01	0.28
No	10.44 ± 6.18		11.97 ± 7.01	
Nausea‐Vomiting				
Yes	10.52 ± 6.55	0.078	13.06 ± 6.94	0.92
No	13.98 ± 7.87		12.92 ± 7.11	
Hemoptysis				
Yes	21.00 ± 8.00	0.036*, ^,a^	18.50 ± 6.55	0.10
No	12.76 ± 7.49		12.72 ± 6.97	
Anosmia				
Yes	10.83 ± 3.54	0.16	16.30 ± 6.27	0.65
No	13.40 ± 7.93		12.43 ± 7.02	
Signs on first visit				
Temperature				
≤37.2	12.22 ± 7.68	0.48	11.47 ± 6.65	0.05
>37.2	13.60 ± 7.73		14.25 ± 7.13	
Respiratory Rate				
≤25	11.21 ± 6.69	<0.0001*, ^,a^	12.01 ± 7.07	<0.0001*, ^,a^
>25	19.50 ± 7.37		17.68 ± 4.48	
SpO_2_				
≤93	15.13 ± 7.71	0.01*, ^,a^	15.31 ± 6.17	<0.0001,*, ^,a^
>93	10.59 ± 6.96		7.62 ± 5.86	
Initial Laboratory findings				
WBC (cell/mm^3^)				
≤10,000	13.74 ± 7.61	0.31	12.50 ± 6.82	0.21
>10,000	11.70 ± 7.88		14.70 ± 7.63	
Lymphocyte count(cell/mm3)				
≤1000	14.63 ± 7.56	0.19	14.26 ± 6.47	0.27
>1000	12.24 ± 7.70		12.47 ± 7.19	
Platelet count				
≤150,000	12.22 ± 6.99	0.48	9.50 ± 4.85	0.012,*, ^,a^
>150,000	13.60 ± 7.99		13.89 ± 7.24	
CRP (mg/L)				
≤90	12.00 ± 7.01	0.27	10.56 ± 6.72	0.001*, ^,a^
>90	14.00 ± 8.09		15.22 ± 6.58	
ESR(mm/h)				
≤60	13.62 ± 9.03	0.80	10.46 ± 6.48	0.032*, ^,a^
>60	13.08 ± 7.36		13.91 ± 7.02	
LDH (units/L)				
≤480	10.81 ± 6.45	0.12	12.00 ± 6.58	0.57
>480	13.84 ± 7.91		13.13 ± 7.11	
AST (units/L)				
≤41	14.18 ± 8.04	0.27	10.92 ± 7.42	0.011*, ^,a^
>41	12.23 ± 7.31		14.58 ± 6.29	
ALT (units/L)				
≤41	14.29 ± 8.05	0.26	11.57 ± 7.55	0.084
>41	12.29 ± 7.35		14.07 ± 6.42	
Underlying disease				
Diabetes				
Yes	12.91 ± 7.57	0.83	12.30 ± 6.65	0.53
No	13.32 ± 7.81		13.27 ± 7.21	
Hypertension				
YES	12.05 ± 7.35	0.45	13.87 ± 7.15	0.37
NO	13.58 ± 7.82		12.50 ± 6.96	
Smoker				
Yes	9.71 ± 4.57	0.08	12.16 ± 5.98	0.77
No	13.55 ± 7.87		13.02 ± 7.11	
CKD				
Yes	8.20 ± 4.32	0.04*, ^,a^	2.33 ± 0.557	<0.001*, ^,a^
No	13.55 ± 7.77		13.31 ± 6.85	
IHD				
Yes	10.83 ± 7.62	0.43	11.88 ± 7.12	0.48
No	13.40 ± 7.71		13.20 ± 7.02	
Outcomes				
Hospitalization				
≤7 days	11.46 ± 6.91	0.016*, ^,a^	12.31 ± 6.74	0.17
>7 days	15.80 ± 8.17		14.44 ± 7.51	
ICU admission				
Yes	16.04 ± 7.33	0.04*, ^,a^	13.30 ± 5.14	0.87
No	12.09 ± 7.60		12.92 ± 7.23	

*Note*: All quantitative data are expressed as mean ± standard deviation.

Abbreviations: ALT, alanine transaminase; AST, aspartate transaminase; CKD, chronic kidney disease; CRP, C‐reactive protein, ESR, erythrocyte sedimentation rate; ICU, intensive care unit; IHD, ischemic heart disease; LDH, lactate dehydrogenase; SpO_2_, oxygen saturation;

* Statistically significant (*p* < 0.05);

^a^ independent *t*‐test or Mann–Whitney *U* test used.

## 4 DISCUSSION

Comorbidities such as arterial HTN, cardiovascular disease, diabetes, and cancer have been recognized as risk factors for severe diseases in the current COVID‐19 pandemic,^12^, ^13^ while the effect of immunodeficiency on COVID‐19 is still controversial. In the present study, we investigated the pulmonary involvement of COVID‐19 in the case‐control group of immunocompromised and immunocompetent patients. Our results showed pulmonary involvement of COVID‐19 was lower among immunocompromised patients compared to immunocompetent patients. Initial symptoms of immunocompromised patients, including dyspnea, were associated with total chest CT score, while, Laboratory findings of immunocompetent patients including platelet count (>150,000), CRP (>90), ESR (>60), and AST (>41) were associated with higher chest CT scores. On the other hand, both immunocompromised and immunocompetent patients with Lower Spo_2_ (≤93), higher respiratory rate (>25), and CKD had higher chest CT scores. Moreover, immunocompromised patients with higher chest CT scores had longer hospitalization and higher ICU admission.

Patchy lesions with confluent shapes and mixed GGO and consolidation patterns were the most prevalent characteristics of lesions. In line with our findings, a previous study on kidney transplant recipients showed the bilateral, diffuse distribution of lesions with GGO and consolidation as the most prevalent characteristics of lesions.^14^ Similar to our findings, another Case‐control study among renal transplant recipients and the general population showed patchy lesions with GGO, and consolidation (with or without other findings) were the most common finding in both groups, but there was no significant difference between groups.^5^ Confirming our findings, a previous descriptive study among the general population also showed that GGO and mixed GGO with consolidation were the most common patterns among COVID‐19 patients.^15^

Our results showed immunocompromised patients generally had less severe pulmonary involvement compared to immunocompetent patients, which might confirm the previous studies suggesting the protective effects of immunosuppression on immunocompromised COVID‐19 patients by preventing the development of cytokine storm and its consequences^16^; Which was previously described how rheumatologic patients might benefit from immunosuppressive therapy.^17^ While clinical data among immunocompromised COVID‐19 patients are scarce, a previous study on kidney transplant patients with pneumonia suggested the low rate of the severe type of disease among kidney transplant recipients might be due to some levels of immunosuppression.^6^ Marcus et al.^18^ also reported out of 1679 primary immunodeficient patients, only 20 patients (1.2%) tested positive for COVID‐19, which is far less common in the general population (~2.5%). Meanwhile, a previous study by Monfared et al.^5^ could not show any significant differences between the severity of radiological findings among immunocompromised (kidney transplant recipients) and non‐immunocompromised patients which might arise from the low number of patients in each group.

Evaluation of total chest CT score among the immunocompromised group suggests severe symptoms like dyspnea and hemoptysis should take better care of, as they show significant severe pulmonary involvement. Confirming our findings, Li et al.^19^ had previously shown clinical factors including dyspnea, cough, and chest pain were risk factors for severe COVID‐19 pneumonia. While hemoptysis is rarely reported in COVID‐19 patients,^1^, ^20^ our results showed this rare symptom is associated with more severe lung involvement in immunocompromised patients. In comparison, the previous study on kidney transplant recipients showed high levels of CRP) > 100 mg/L(, interleukin‐6 (>65 ng/L), high‐sensitivity troponin I (>30 ng/L), and d‐dimer (>960 ng/mL) were significantly associated with severe disease and mortality,^21^ our laboratory findings were not distinctive for more pulmonary involvement in immunocompromised patients.

Initial symptoms of immunocompetent patients were not distinctive for severe lung involvement, while laboratory findings were highly associated with more pulmonary involvement scores, as higher platelet count, ESR, CRP, and AST were associated with higher chest CT scores, which might also be justified by normal reaction of the immune system to COVID‐19 infection by initiating the cytokine storm.^22^ Similar to our findings, Zhang et al. reported higher WBC count, CRP, and ESR were associated with higher chest CT scores,^23^ confirming our findings, a meta‐analysis investigating the association of inflammatory markers with the severity of COVID‐19 showed increased levels of CRP, Interleukin‐6, ESR, Serum amyloid A, Procalcitonin and serum ferritin are an indicator for the severity of COVID‐19.^24^

Initial higher respiratory rate and lower Spo_2_ were a reliable predictor of higher pulmonary involvement in both groups; meanwhile, CKD was also associated with higher chest CT scores in both groups. Similar to our findings, previous studies had already emphasized the relation between low capillary blood oxygen and higher chest CT scores.^25^, ^26^ Confirming our findings, several systematic reviews^27^, ^28^, ^29^ previously reported patients with chronic or acute kidney disease developed more severe COVID‐19 disease or outcomes, whereas our study is, to the best of our knowledge, the first study showing CKD can worsen the disease process of lung injury in immunocompromised patients.

Further analysis showed longer hospital stays and ICU admission were associated with higher chest CT scores of immunocompromised patients, while immunocompetent patients had no meaningful association with their hospitalization time and ICU admission with their chest CT scores. In line with our results, previous studies showed higher chest CT scores were predictive of longer hospital stays and ICU admission^10^, ^30^, ^31^; while these studies showed this correlation in the general population, our results only demonstrated this correlation among immunocompromised patients.

Due to the lack of immune response in immunocompromised patients, we investigated the pulmonary involvement by chest CT score in the case‐control group of immunocompromised and immunocompetent patients. Our study suggests due to the lower immune response of immunocompromised patients, laboratory and inflammatory markers cannot show the extent of pulmonary involvement, while the patient's initial signs and symptoms, including dyspnea, hemoptysis, higher RR, and lower Spo_2_, could show the severity of lung involvement.

### 4.1 Limitations

The immunocompromised group in our study consisted wide range of patients with different causes of immunodeficiency, which might affect our results. We had no microbiological confirmation for every patient, and some of the patients were included according to their clinical and imaging findings. Due to the Retrospective nature of the current case‐control study, we recommend further prospective studies to investigate the disease course and pulmonary involvement in each specific group of immunocompromised patients.

## 5 CONCLUSIONS

Initial clinical findings, including dyspnea, hemoptysis, higher RR, and lower Spo_2_, were better predictors of pulmonary involvement among immunocompromised patients than laboratory findings, and due to lower immune response in immunocompromised patients, pulmonary involvement is less severe than immunocompetent counterparts.

## AUTHOR CONTRIBUTIONS

Abdolkarim Haji Ghadery contributed to the analysis, interpretation of data, and writing the manuscript. Ladan Abbasian contributed to the acquisition of data and the design of the study. Fatemeh Jafari contributed to the acquisition of data. Niloofar Ayoobi Yazdi contributed to the conception and design of the study and revising the manuscript. Zahra Ahmadinejad contributed to the conception and design of the study. All authors reviewed and approved the submitted version of the manuscript.

## CONFLICT OF INTEREST STATEMENT

The authors declare no conflict of interest.

## ETHICS STATEMENT

The Institutional Review Board (IRB) of the Tehran University of Medical Sciences examined and approved the study's ethical requirements (IR.TUMS.VCR.REC.1399.053). The study was conducted in accordance with the declaration of Helsinki guidelines, and informed consent was obtained from all participants or their legal guardians. The clinical and laboratory information of the study patients is presented in the journal of Immunity, Inflammation and Disease.

## Data Availability

The datasets generated and/or analyzed during the current study are not publicly available but are available from the corresponding author at a reasonable request.
